# Lack of effect of high-protein vs. high-carbohydrate meal intake on stress-related mood and eating behavior

**DOI:** 10.1186/1475-2891-10-136

**Published:** 2011-12-12

**Authors:** Sofie G Lemmens, Eveline A Martens, Jurriaan M Born, Mieke J Martens, Margriet S Westerterp-Plantenga

**Affiliations:** 1 Top Institute Food and Nutrition, Wageningen, The Netherlands; 2 Department of Human Biology, Maastricht University, Maastricht, The Netherlands

**Keywords:** Macronutrients, stress, reward, disinhibition

## Abstract

**Background:**

Consumption of meals with different macronutrients, especially high in carbohydrates, may influence stress-related eating behavior. We aimed to investigate whether consumption of high-protein vs. high-carbohydrate meals influences stress-related mood, food reward, i.e. 'liking' and 'wanting', and post-meal energy intake.

**Methods:**

Participants (n = 38, 19m/19f, age = 25 ± 9 y, BMI = 25.0 ± 3.3 kg/m^2^) came to the university four times, fasted, once for a stress session receiving a high-protein meal, once for a rest session receiving a high-protein meal, once for a stress session receiving a high-carbohydrate meal and once for a rest session receiving a high-carbohydrate meal (randomized cross-over design). The high-protein and high-carbohydrate test meals (energy percentage protein/carbohydrate/fat 65/5/30 vs. 6/64/30) matched for energy density (4 kJ/g) and daily energy requirements (30%). Stress was induced using an ego-threatening test. Pre- and post-meal 'liking' and 'wanting' (for bread, filling, drinks, dessert, snacks, stationery (non-food alternative as control)) was measured by means of a computer test. Following the post-meal 'wanting' measurement, participants received and consumed their wanted food items (post-meal energy intake). Appetite profile (visual analogue scales), mood state (Profile Of Mood State and State Trait Anxiety Inventory questionnaires), and post-meal energy intake were measured.

**Results:**

Participants showed increased feelings of depression and anxiety during stress (P < 0.01). Consumption of the test meal decreased hunger, increased satiety, decreased 'liking' of bread and filling, and increased 'liking' of placebo and drinks (P < 0.0001). Food 'wanting' decreased pre- to post-meal (P < 0.0001). The high-protein vs. high-carbohydrate test meal induced lower subsequent 'wanting' and energy intake (1.7 ± 0.3 MJ vs. 2.5 ± 0.4 MJ) only in individuals characterized by disinhibited eating behavior (factor 2 Three Factor Eating Questionnaire, n = 16), during rest (P ≤ 0.01). This reduction in 'wanting' and energy intake following the high-protein meal disappeared during stress.

**Conclusions:**

Consumption of a high-protein vs. high-carbohydrate meal appears to have limited impact on stress-related eating behavior. Only participants with high disinhibition showed decreased subsequent 'wanting' and energy intake during rest; this effect disappeared under stress. Acute stress overruled effects of consumption of high-protein foods.

**Trial registration:**

The study was registered in the Dutch Trial Register (NTR1904). The protocol described here in this study deviates from the trial protocol approved by the Medical Ethical Committee of the Maastricht University as it comprises only a part of the approved trial protocol.

## Background

Recent human studies have shown a possible relationship between stress and the increased prevalence of obesity [1-4]. In a previous study we showed that overweight individuals with abdominal adiposity showed stress-induced food intake in the absence of hunger, resulting in an increased energy intake [5]. Moreover, Rutters et al. showed that acute psychological stress leads to eating in the absence of hunger, especially in vulnerable individuals characterized by disinhibited eating behavior [6]. The food choice in stress is often shifted towards sweet and fat foods, possibly because they are perceived as highly rewarding [5-8]. Consumption of those 'comfort foods' may be a way to cope with stress [9]. However, several endocrinological studies showed that some of these preferred or highly rewarding foods, namely foods high in carbohydrates, may not reduce stress but even increase stress, i.e. hypothalamus pituitary adrenal (HPA) axis activity, represented by cortisol concentrations [10-12]. Moreover, the risk is that chronic stress combined with a high-fat, high-carbohydrate diet may lead to abdominal obesity [9].

The regulation of food intake and energy homeostasis involves, besides 'hunger' and 'satiety' signals, factors such as food reward, environmental cues, and cognitive factors [13,14]. In some situations, e.g. stress or the abundance of palatable foods, the food reward system may overrule and promote eating in the absence of hunger and consequently in the long-term a positive energy balance [13,15,16]. According to the incentive salience theory, it is hypothesized that the process of reward consists of two components controlled by different brain mechanisms, i.e. 'liking' and 'wanting' [17]. 'Liking', under control of opioids, is the hedonic or affective component and refers to the pleasure derived from oro-sensory stimulation of food [18,19]. 'Wanting', under control of dopamine, is the motivational incentive component and refers to appetite or craving or the motivation to obtain food [17-21]. Evidence for the involvement of the reward system in stress-induced eating can be found in both rodent and human studies [5,22-30].

Since the macronutrient composition of a meal may influence HPA axis activity physiologically [10-12], we hypothesized that consumption of isocaloric meals with a different macronutrient composition, i.e. a high-protein vs. high-carbohydrate meal, may also influence the psychological stress response differently. Moreover it may affect the rewarding value of food, i.e. 'liking' and 'wanting', and the stress-induced food choice and subsequent food intake.

The macronutrient composition of a meal may also influence the mood response to stressors [31]. Increases in negative mood in response to stressors can lead to greater food intake [6,32]. Consumption of foods that improve the stress-induced mood state may prevent further intake of energy-dense foods. A study by Firk and Markus showed that intake of tryptophan-rich hydrolyzed protein increased positive mood to acute stress [31]. Based upon this latter study, and the endocrinological studies by Lacroix et al., Martens et al., and Vicenatti et al. [10-12] showing that a high-protein meal prevents increases in stress cortisol levels, we hypothesized that a high-protein meal, in contrast to a high-carbohydrate meal, may reduce post-meal energy intake during stress. Therefore we investigated whether the consumption of a high-protein vs. high-carbohydrate meal influences the stress-induced psychological mood response, the rewarding value of food, i.e. 'liking' and 'wanting', and the stress-induced food choice and food intake. Moreover, we investigated whether consumption of those meals would affect in particular overweight individuals with abdominal adiposity and individuals characterized by disinhibited eating behavior, as it has been shown that those individuals are more vulnerable to stress-induced eating [5,6].

## Methods

### Participants

Thirty-eight healthy Caucasian participants (19 men and 19 women; age 25 ± 9 y (mean ± SD, range 18-51 y)) with a body mass index (BMI) of 25.0 ± 3.3 kg/m^2 ^(mean ± SD, range 20.3-31.2 kg/m^2^) participated in this study. Based upon the study by Lemmens et al. [5], power analysis showed that with an α of 0.0125 (taking into account the Bonferroni correction for multiple testing) and β of 0.10 (power = 1-β = 0.90), at least 31 participants were needed. They were recruited by advertisements in local newspapers and on notice boards at the university. Participants underwent an initial screening including measurement of body weight, height, waist circumference and hip circumference, and completed a questionnaire related to health, use of medication, physical activity, and eating behavior. Inclusion criteria comprised BMI 20-30 kg/m^2^, both genders, no use of medication (except contraception), no food allergies, no dietary restrictions, and not pregnant or breast-feeding. Regarding overweight participants only participants with abdominal adiposity were included, as chronic stress has been associated with visceral fat accumulation and obesity [1,33,34]. Abdominal adiposity was defined as having a waist circumference of ≥ 80 cm in women and ≥ 94 cm in men [35].

Eating behavior was analyzed using a validated Dutch translation of the Three Factor Eating Questionnaire (TFEQ) which measures three components: 'cognitive restraint of eating' (factor 1, F1), 'disinhibition of restraint' (factor 2, F2), and 'hunger' (factor 3, F3) [36]. On the basis of the median for the TFEQ scores in the south of the Netherlands, participants were characterized as unrestraint when dietary restraint scores were < 9, and as restraint when scores were ≥9. Participants were characterized as having low disinhibition when disinhibition scores were < 5, and as having high disinhibition when scores were ≥5 [37].

All participants gave written informed consent and the study was approved by the Medical Ethical Committee of the Maastricht University.

### Study design

The study was conducted in a randomized cross-over design. All participants came to the university four times, in a fasted state (for at least 8 h), between 08:00 and 9:00 AM. They came once for a stress session receiving a high-protein meal, once for a rest session receiving a high-protein meal, once for a stress session receiving a high-carbohydrate meal and, once for a rest session receiving a high-carbohydrate meal. The order of the four conditions was randomized across the participants to prevent any order effects. The four test sessions were at least one week apart.

**Figure 1:**
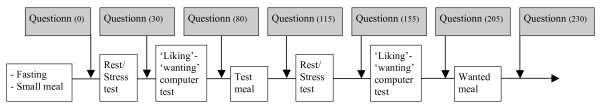
**Schematic overview of the study design**. Numbers in brackets represent the time points (in minutes) at which data were collected or tasks were completed; 'Question': questionnaires.

Figure 1 gives a schematic overview of the study design. After arrival at the university, participants were seated in the laboratory and remained seated throughout the experiment. Each test session participants received 50 g of yoghurt ('Campina magere yoghurt naturel', 84 kJ, energy percentage protein/carbohydrate/fat (En% P/C/F) 53/44/2) to prevent large hunger feelings. The test sessions started two hours later, to give the participants the chance to adapt to the laboratory environment.

An ego-threatening computer test containing elements of an IQ-test was used to create the stress vs. rest conditions in participants [6,38,39]. Two versions of this psychological test were used: a difficult stress version with not enough time to solve the assignments and an easier control version with enough time to solve the assignments. The psychological test was an updated version of the test used by Rutters et al. and Born et al. [6,30].

Following this psychological test (Figure 1, 'Rest/Stress test') food reward, i.e. 'liking' and 'wanting', was measured by means of a computer test, described and validated by Lemmens et al. [40]. During the 'liking' part of the computer test, participants had to indicate their relative preference of paired items within and between six categories: bread, filling, drinks, dessert, snacks, and stationery (non-food alternative as control). During the 'wanting' part, participants had to work to earn items by playing memory games for each of the same six categories.

After completing the 'liking' and 'wanting' computer test participants were offered the test meal (lunch), which was either a high-protein meal or a high-carbohydrate meal, and which had to be consumed entirely. Participants received an amount corresponding to 30% of their daily energy requirements.

Following test meal consumption, participants completed the psychological test and the 'liking' and 'wanting' computer test again. Subsequently, participants received and consumed their wanted food items (Figure 1, 'Wanted meal', post-meal energy intake), which were chosen by means of the 'wanting' part of the computer test.

The psychological 'Rest/Stress test' and the 'liking' and 'wanting computer test were completed pre and post test meal to be able to measure effects of stress on food choice and food intake in hunger as well as in the satiated state. During the rest session participants completed twice the control (rest) version of the psychological test, and during the stress session they completed twice the stress version of the psychological test.

To investigate whether the stress condition inflicted psychological changes, we used Profile Of Mood State (POMS) and State Trait Anxiety Inventory (STAI) questionnaires. One hundred unit visual analogue scales (VAS; in mm) were used to assess the appetite profile. Questionnaires were collected seven times per test session.

All women were tested in the follicular phase, as it has been shown that women have a higher spontaneous energy intake in the luteal phase compared with the follicular phase [11,41].

### Test meals

The test meal was either a high-protein lunch (En% P/C/F 65/5/30) or a high-carbohydrate lunch (En% P/C/F 6/64/30). Both meals were isocaloric and matched for energy density: 4 kJ/g. The amount of the meals that was given to the participants corresponded to 30% of their daily energy requirements (DER). For each participant the DER were calculated by multiplying the basal metabolic rate (BMR) by the appropriate physical activity factor (1.5-1.8, derived from the screening questionnaire, [42]). The BMR (kcal/day) was calculated according to the equation of Harris-Benedict [43].

The high-protein meal consisted of a salad (iceberg lettuce, cucumber, mushroom, and sunflower oil), Gouda cheese, salami, and a strawberry protein shake (protein source: milk protein). The high-carbohydrate meal consisted of a salad (iceberg lettuce, cucumber, green pepper, and sunflower oil), savory cheese biscuits and TUC bacon biscuits, and a strawberry carbohydrate shake (type of carbohydrate: dextrin maltose). In both meals the shakes represented 47 En% of the total meal.

During the screening session participants had to taste and rate the food items, which would have to be consumed on the test days, for subjective liking (VAS), in order to check whether all food items were acceptable. All food items scored more than 60 mm on a 100-mm VAS.

### Questionnaires

One hundred unit VAS (mm) were used to assess the appetite profile. The scales were anchored with 'not at all' at one end and 'extremely' at the other end, and combined with questions on feelings of hunger, thirst, fullness, satiety, and desire to eat, and on subjective liking and wanting of the test meals.

Mood states were assessed using a modified version of the Dutch translation of the POMS [44]. This questionnaire contains 35 adjectives that are rated on a five-point scale and is divided into five subscales (depression, tension, confusion, fatigue, and anger). The Dutch translation of the state scale of the STAI questionnaire was used to measure state anxiety [45]. Participants had to rate 20 statements on how they felt at that moment on a four-point scale. An increase in POMS and STAI scores is associated with a worsening in mood.

The VAS (for hunger, thirst, fullness, satiety and desire to eat), POMS, and STAI questionnaires were completed seven times throughout the test sessions at 0, 30, 80, 115, 155, 205 and 225 minutes (Figure 1). The VAS on subjective liking and wanting of the test meals were completed pre and post test meal consumption (at 80 and 115 minutes).

### 'Liking' and 'wanting' computer test

The computer test described and validated by Lemmens et al. [40] was used to measure the rewarding value, i.e. 'liking' and 'wanting', for 72 items divided in six categories: bread, filling, drinks, dessert, snacks, and stationery (non-food alternative as control). Each category contained 12 items. The 72 items were presented as photographic stimuli on a computer screen (13-inch Mac Book, Apple, Cupertino, USA).

The computer test contained two parts, a 'liking' part and a 'wanting' part. Both the 'liking' and 'wanting' tasks assessed 'liking' respectively 'wanting' for the same food and stationery items. During the 'liking' part, participants had to indicate their relative preference of paired items within and between the six categories. This resulted in a relative ranking of 'liking' of the items per category and of the categories.

During the 'wanting' part, participants had to work to earn items by playing memory games. For each category of items participants played a five by five memory game (12 pairs of items) followed by the indication of the items participants wanted to acquire at that moment. The more pairs of items were found in the memory game, the more randomly selected items were offered to choose from afterwards, e.g. if eight pairs of items would be found in the memory game of the snacks category, then eight randomly selected snacks would be offered to choose from. Participants could choose zero, one or two items per category. They were instructed to choose the items while keeping in mind that all the chosen items would be offered to them and had to be eaten completely. The chosen items obtained a score equal to the number of pairs of items found in the memory game, representing the motivation or workload for the chosen items. Items not chosen obtained a score of zero. Per category the sum of the scores of the items was calculated and represented the 'wanting' score for each category. During the screening session participants were tested on their ability to play a five by five memory game within two minutes.

All the food items chosen by means of the 'wanting' part of the post test meal computer test were offered to the participants at a fixed amount, which was described to the participants beforehand, and food items were eaten completely (Figure 1, 'wanted meal'). Total energy content (post-meal energy intake) and macronutrient composition of the consumed wanted food items was calculated.

### Statistics

Data were analyzed using StatView 5.0 (SAS Institute Inc., Cary, NC, USA). Unpaired Student's t-tests were used to analyze differences in participant characteristics between men and women. Factorial ANOVA with repeated measures was used to study the effects between participant groups (men vs. women, overweight vs. normal weight, high vs. low disinhibition) of the conditions of stress vs. rest and of high-protein vs. high-carbohydrate, and of time (pre and post test meal), on data of the questionnaires (VAS, POMS, STAI), on data of the 'liking' and 'wanting' computer test, and on post-meal energy intake. Paired and unpaired Student's t-tests were used as Post hoc analyses for significant interactions. Areas under the curve (AUC) for questionnaire data were calculated using the trapezoid method. All tests were two-sided and differences were considered significant at P < 0.05. Values are expressed as mean ± standard error of the mean (SEM), unless stated otherwise.

Neither gender differences (men vs. women), nor differences according to weight status (overweight vs. normal weight) were detected concerning possible conditional effects of stress vs. rest and of high-protein vs. high-carbohydrate on data of the questionnaires (POMS, STAI, VAS), on data of the 'liking' and 'wanting' computer test, and on post-meal energy intake. However, an effect of disinhibited eating behavior was detected for some of the measured variables. Therefore, results were presented firstly for all participants together and secondly for individuals characterized by high vs. low disinhibited eating behavior (high F2 score: n = 16, 6 men and 10 women; low F2 score: n = 22, 13 men and 9 women).

## Results

### Participant characteristics

No significant differences were shown between men and women concerning age, BMI, hip circumference, and disinhibition scores (Table 1). Women had higher scores for dietary restraint and feeling of hunger when compared with men (P < 0.05). Men had a larger height, body weight, and waist circumference when compared with women (P < 0.05).

**Table 1:** Characteristics of men and women.

	Men (n = 19)	Women (n = 19)	P^a^
Age (y)	25.6 ± 8.6	24.9 ± 9.3	n.s.
Height (cm)	180.2 ± 7.7	168.6 ± 6.4	<.0001
Body weight (kg)	80.1 ± 8.8	71.6 ± 9.4	<.01
BMI (kg/m^2^)	24.8 ± 3.4	25.2 ± 3.2	n.s.
Waist circumference (cm)	86.4 ± 9.7	79.9 ± 9.9	<.05
Hip circumference (cm)	103.7 ± 5.5	105.5 ± 5.1	n.s.
Dietary restraint score	4.7 ± 3.7	7.5 ± 4.0	<.05
Disinhibition score	3.9 ± 1.4	5.1 ± 2.9	n.s.
Feeling of hunger score	3.1 ± 2.3	5.6 ± 3.4	<.01

Values are mean ± SD

^a^P-value: differences between men and women (unpaired Student's t-tests)

n.s. = non-significant

Participants characterized by high vs. low disinhibited eating behavior differed only in the TFEQ score for disinhibition and feeling of hunger (F2 score 6.4 ± 2.2 vs. 3.0 ± 0.8, F3 score 5.8 ± 4.0 vs. 3.3 ± 1.9 (mean ± SD), P < 0.02). Their BMI did not differ (25.7 ± 3.7 kg/m^2 ^vs. 24.5 ± 2.9 kg/m^2^).

### Stress parameters

Scores of POMS and STAI questionnaires showed higher feelings of depression (POMS ΔAUC = +210.8 ± 76.4 × minute^-1^, P < 0.01), tension (POMS ΔAUC = +227.7 ± 74.0 × minute^-1^, P < 0.01), confusion (POMS, ΔAUC = +180.7 ± 71.7 × minute^-1^, P < 0.02), anger (POMS ΔAUC = +211.5 ± 74.3 × minute^-1^, P < 0.01), and anxiety (STAI ΔAUC = +415.5 ± 126.8 × minute^-1^, P < 0.01), during the stress vs. rest sessions (ANOVA repeated measures: AUC of POMS and STAI scores stress vs. rest × high-protein vs. high-carbohydrate, main effect of stress), indicating that the applied stressor was effective in inducing psychological stress, regardless of the dietary condition. Consumption of the high-protein vs. high-carbohydrate meal did not affect feelings of depression, tension, anger and anxiety differently (ANOVA repeated measures: change in POMS and STAI scores pre- to post-meal stress vs. rest × high-protein vs. high-carbohydrate, P > 0.1).

There were no differences in POMS and STAI scores between participants with high vs. low disinhibition, in all conditions (AUC and per time point).

### Appetite profile

The fasted state was confirmed by low satiety and fullness scores (11.8 ± 2.5, 9.6 ± 1.9 mmVAS), and high hunger, 'desire to eat', and thirst scores (80.6 ± 2.6, 83.9 ± 2.2, 68.1 ± 3.7 mmVAS). Consumption of the test meal resulted in an increase in satiety and fullness scores (Δ = -63.2 ± 4.6, -69.9 ± 3.7 mmVAS, P < 0.001), and a decrease in hunger, 'desire to eat', and thirst scores (Δ = +67.8 ± 3.3, +68.5 ± 3.3, +33.8 ± 4.3 mmVAS, P < 0.001). Conditions of stress vs. rest and of high-protein vs. high-carbohydrate did not affect feelings of hunger, thirst, desire to eat, satiety, and fullness (AUC and per time point). VAS scores for the appetite profile parameters hunger, 'desire to eat', thirst, satiety, and fullness did not differ between participants with high vs. low disinhibition, in all conditions (AUC, per time point, and change in score pre to post test meal consumption).

Consumption of the test meals decreased VAS liking and wanting scores for the test meals (average liking scores pre- and post-meal: 53.5 ± 3.7, 43.4 ± 4.0 mmVAS; P < 0.001; average wanting score pre- and post-meal: 65.3 ± 4.3, 8.7 ± 2.0 mmVAS;). Conditions of stress vs. rest and of high-protein vs. high-carbohydrate did not influence liking (VAS) of the test meals pre- and post-meal, confirming that the meals were comparably liked. The condition of stress vs. rest did not influence wanting (VAS) of the test meals pre- and post-meal, though during stress the decrease in wanting pre- to post-meal was larger in the high-protein condition compared with the high-carbohydrate condition (P = 0.03).

Liking (VAS) of the test meals did not differ between participants with high vs. low disinhibition, in all conditions. Wanting (VAS) did not differ either, except for post-meal wanting of the high-protein meal during stress, which was lower in participants with high vs. low disinhibition (P < 0.01).

### Computer test

**Figure 2:**
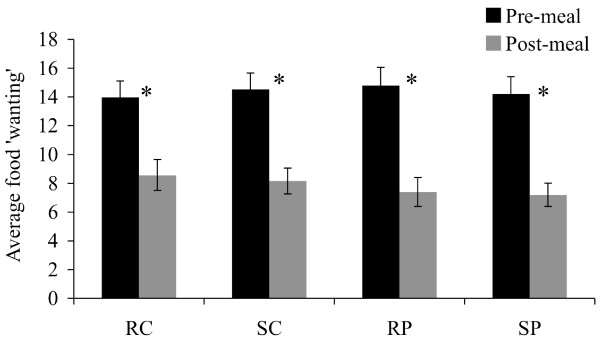
**Average food 'wanting' score (mean ± SEM) pre- and post-meal**. n = 38; Average food 'wanting': 'wanting' for items from all the five food categories taken together; Four conditions: RC = rest-carbohydrate, SC = stress-carbohydrate, RP = rest-protein, SP = stress-protein; *P < 0.0001.

'Liking' between categories, average food 'wanting' ('wanting' for food items from all the five food categories taken together) and 'wanting' per category were not influenced by the conditions of stress vs. rest and high-protein vs. high-carbohydrate. There was an overall effect of time on 'liking' for bread, filling, drinks and placebo, and on average food 'wanting and 'wanting' per category (P < 0.0001). Consumption of the test meal decreased ranking of 'liking' of bread and filling and increased ranking of 'liking' of placebo and drinks, in all conditions (P < 0.0001). Consumption of the test meal decreased average food 'wanting' in all conditions (P < 0.0001, Figure 2). More specifically, there was a pre- to post-meal decrease in 'wanting' for bread, filling, drinks, dessert, and snacks (P < 0.04).

**Figure 3:**
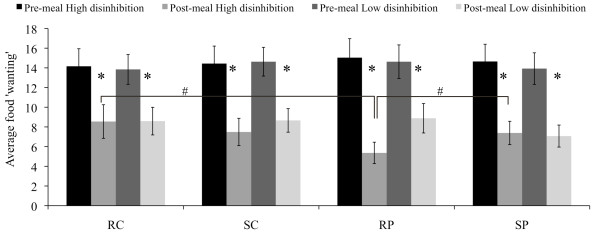
**Average food 'wanting' score (mean ± SEM) pre- and post-meal, for participants with high vs. low disinhibition**. n = 38 (16 vs. 22); Average food 'wanting': 'wanting' for items from all the five food categories taken together; Four conditions: RC = rest-carbohydrate, SC = stress-carbohydrate, RP = rest-protein, SP = stress-protein; *P < 0.01 for differences pre- to post-meal, #P < 0.02.

An effect of disinhibited eating behavior was detected for average food 'wanting': there was an interaction effect of post-meal food 'wanting' stress vs. rest × high-protein vs. high-carbohydrate × high vs. low disinhibition (P < 0.04, ANOVA repeated measures). The high-protein meal, vs. high-carbohydrate meal, induced lower subsequent average food 'wanting' in individuals with high disinhibition, during the rest condition (P < 0.01, Figure 3). Moreover, participants with high disinhibition showed higher average food 'wanting' in the stress vs. rest condition following the high-protein meal (P < 0.02, Figure 3).

### Energy and macronutrient intake

**Figure 4:**
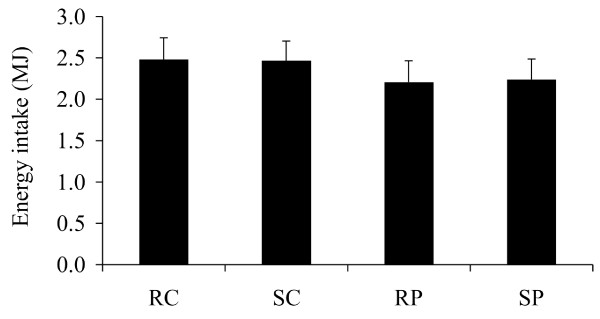
**Post-meal energy intake (MJ, mean ± SEM, 'wanted meal')**. n = 38; Four conditions: RC = rest-carbohydrate, SC = stress-carbohydrate, RP = rest-protein, SP = stress-protein.

**Figure 5:**
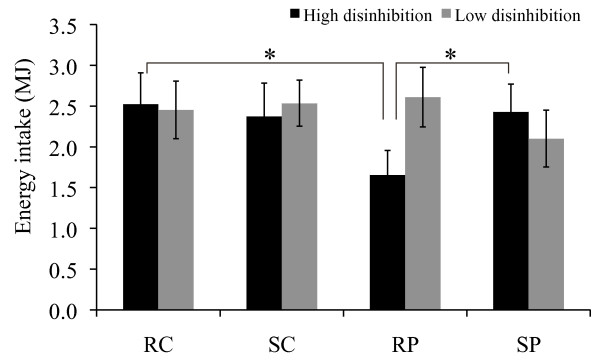
**Post-meal energy intake (MJ, mean ± SEM, 'wanted meal') for participants with high vs. low disinhibition**. n = 38 (16 vs. 22); Four conditions: RC = rest-carbohydrate, SC = stress-carbohydrate, RP = rest-protein, SP = stress-protein; *P ≤ 0.01.

Post-meal energy intake of the wanted food items ('wanted meal') was not influenced by the conditions of stress vs. rest and high-protein vs. high-carbohydrate **(**Figure 4). An effect of disinhibited eating behavior was detected for post-meal energy intake: there was an interaction effect of post-meal energy intake stress vs. rest × high-protein vs. high-carbohydrate × high vs. low disinhibition (P = 0.01, ANOVA repeated measures). The high-protein meal, vs. high-carbohydrate meal, induced lower subsequent energy intake in individuals with high disinhibition, during rest (P < 0.01), but not during stress **(**Figure 5). Moreover, participants with high disinhibition showed higher energy intake in the stress vs. rest condition following the high-protein meal (P = 0.01, Figure 5).

Analyzing the amount of carbohydrates, fat, and proteins consumed for the 'wanted meal', showed that consumption of the high-protein meal, vs. high-carbohydrate meal, induced lower subsequent intake of carbohydrates, fat, as well as proteins in individuals with high disinhibition, during the rest condition (P < 0.05), but not during the stress condition.

## Discussion

The main objective of this study was to investigate possible effects of consumption of a high-protein vs. high-carbohydrate meal on the stress-induced psychological mood response, the rewarding value of food, i.e. 'liking' and 'wanting', and the stress-induced food choice and subsequent food intake.

The conditions of the satiated vs. hungry state and of the stress vs. rest condition were confirmed. Consumption of isocaloric meals with different macronutrient contents did not influence the stress-induced psychological mood response. This contrasts the study by Firk and Markus [31] showing that intake of tryptophan-rich hydrolyzed protein increased positive mood to acute stress. Moreover, consumption of isocaloric meals with different macronutrient contents did not influence the rewarding value of food, and the subsequent food choice and food intake differently.

Based upon the endocrinological studies by Lacroix et al., Martens et al., and Vicenatti et al. [10-12], showing that a high-protein meal prevents increases in stress cortisol levels, we expected that a high-protein meal, in contrast to a high-carbohydrate meal, may reduce subsequent eating during stress. The anticipated effect on stress and on food intake in the stress condition stayed away. Similarly, based upon the studies by Martens et al. and Vicenatti et al. [11,12], we expected that consumption of a high-carbohydrate meal, in contrast to a high-protein meal, would increase stress-induced eating. Nevertheless, we did not observe increased eating following a high-carbohydrate meal, under stress. All in all, it appears that the macronutrient intakes in our study are not able to affect the effects of stress, neither exaggerating nor diminishing food intake while not being hungry. Moreover there was no effect of weight status (overweight vs. normal weight) on stress-induced post-meal energy intake, which is in contrast to what has been shown in a previous study [5]. It might be that the type of acute stressor used in the laboratory context is too light to elicit a response regarding food intake behavior.

However, a conditional effect of high-protein vs. high-carbohydrate was detected only in participants characterized by disinhibited eating behavior. Average food 'wanting' and energy intake was lower following the high-protein meal, compared with the high-carbohydrate meal, during rest. This reduction in average food 'wanting' and energy intake following the high-protein meal disappeared during stress. Post-meal wanting (VAS) of the high-protein meal during stress, was lower in participants with high vs. low disinhibition. It seems that during stress the high-protein meal was less rewarding for participants with high disinhibition, compared with participants with low disinhibition. This may explain why during stress, in contrast to rest, the high-protein meal did not induce a decreased post-meal 'wanting' and energy intake of other food items in participants with high disinhibition. Previous research has shown that participants with high disinhibition may be vulnerable to ego-threatening stress, leading to an increased energy intake [6,46]. Disinhibited eating behavior can be a possible risk factor for overweight and obesity [47]. Results of our study show a higher average food 'wanting' and energy intake in participants with high disinhibition in the stress vs. rest condition following the high-protein meal. Acute stress overruled the effect of the reduced 'wanting' and energy intake following consumption of a high-protein vs. high-carbohydrate meal.

Why participants with high disinhibition show lower food 'wanting' and energy intake following a high-protein meal, compared with a high-carbohydrate meal, during rest, remains questionable. It cannot be explained by the appetite profile parameters, as they were not affected by the conditions of stress vs. rest and of high-protein vs. high-carbohydrate. It is known from literature that protein is the most satiating macronutrient, and that high-protein meals are more satiating than high-carbohydrate meals [48]. However, our results showed no greater feelings of satiety in the high-protein vs. high-carbohydrate condition. A possible explanation might be that the morning consumption of 50 g of yoghurt was relatively high in protein, and due to this high protein content the lower protein intake and higher carbohydrate intake two hours later might not have resulted in a difference in feelings of satiety at that moment.

To summarize, to our knowledge, this is the first study investigating possible effects of macronutrients, i.e. high-protein vs. high-carbohydrate, on the rewarding value of food, i.e. 'liking' and 'wanting', and on the stress-induced food choice and subsequent food intake. In our everyday life stress is a pervasive factor, and the development of functional foods, able to regulate the stress response, would be helpful to improve or maintain quality of life [49]. However, foods with the macronutrient contents used in our study seem ineffective in regulating the psychological stress response, the rewarding value of food, and the stress-induced food choice and food intake.

## Conclusions

In conclusion, consumption of a high-protein meal, compared with a high-carbohydrate meal, appears to have limited impact on stress-related eating behavior. Only participants with high disinhibition showed decreased subsequent 'wanting' and energy intake during rest; this effect disappeared under stress. Acute stress overruled food intake suppression effects of consumption of high-protein foods.

## List of Abbreviations

AUC: Area under the curve; BMR: Basal metabolic rate; BMI: Body mass index; DER: Daily energy requirements; En%P/C/F: Energy percentage protein/carbohydrate/fat; HPA axis: Hypothalamus pituitary adrenal axis; POMS: Profile Of Mood State; SEM: Standard error of the mean; STAI: State Trait Anxiety Inventory; TFEQ: Three Factor Eating Questionnaire; VAS: Visual analogue scale.

## Competing interests

The authors declare that they have no competing interests.

## Authors' contributions

The study was designed by MSWP and SGL. SGL together with EAM (supervised by MSWP) carried out the study and collected the data. JMB updated the psychological computer test to create the stress vs. rest condition in participants. SGL analyzed the data and wrote the largest part of the manuscript. JMB and MJM reviewed the manuscript. All authors read and approved the final manuscript.
